# Abstract of Proceedings of the Buffalo Medical Association

**Published:** 1867-01

**Authors:** Thos. M. Johnson

**Affiliations:** Sec’y.


ART. III—*Abstract of Proceedings of the Buffalo Medical Association.*

TUESDAY EVENING, December 4, 1866.

Dr. Gleason in the Chair. Present—Drs. Samo, Gay, Smith, Gleason, Miner, Little, Mackey, Cronyn, Wyckoff and Johnson. The minutes of the last meeting were read and adopted.

DR. GAY's treatment of gall stones by oleum olive. The Doctor made a few remarks upon the importance of these cases, and reported the case of a lady 40 years of age, who had been treated by several physicians of high repute, without relief. Gave her at bedtime oleum olive ℥viii. Repeated the dose early in the morning, and soon after gave an ordinary dose of oleum ricini. Evacuation of the bowels soon followed, when several small gall stones were evacuated.

Second case. A man from Ohio, suffering very severely from the third attack. Gave morphine by hypodermic injection to allay the pain. Gave at bedtime oleum olive ℥viii, and repeated the dose at three o'clock in the morning, followed by podophyllin as a cathartic. Two or three evacuations of the bowels followed, when

39 or 40 small gall stones, saturated with oil, were passed, several of which were presented to the Association for examination.

Dr. G. regards the above the best method of treatment for this troublesome disease.

Dr. Miner said, that to say that the substances presented by Dr. Gay are true biliary calculi, is more than their appearance would warrant; they are soft and pliable, and not hard like gall stones. Dr. Gay suggests that the oil is almost as much a specific in this disease, as sulphur is for scabies or mercury for syphilis. It is a lubricant, slightly cathartic, and probably nothing more. Such concretions as these before us often form in the intestinal canal and pass off without the aid of medicine, that they should pass off with the oil is not remarkable. It is not clear to me that they were removed any more by sweet oil than they would have been by any other oil, neither is it probable to my mind that they are true billiary calculi, but if it should even be shown that they are of such nature, I am still unwilling to attribute to the oil any special influence in their removal.

Dr. Gay: I do not consider the oil cathartic. In the cases mentioned I gave a cathartic after giving the oil. In the second case reported the patient was suffering severely from the third attack, and had all the symptoms of the disease. After taking the oil he passed sixty or seventy of these gall stones. They looked unlike feces. They were saturated with oil and softened; the pain with the other symptoms was relieved by the treatment. I believe that this will become *the* treatment for gall stones. Have for a long time given olive oil as a laxative for children, but found that a half pint was not laxative in the cases above mentioned. I report these cases for the purpose of calling out remarks from others.

Dr. Miner: The Doctor speaks as confirmatory of what he has said before, that the pain and other symptoms subsided after the passage of the "gall stones." I have always considered the diagnosis of such cases very doubtful, until the concretions which had passed the billiary duct were found, and if pain subsided after the evacuation of the bowels it would indicate colic, rather than passage of billiary calculi.

Dr. Gay did not make use of the oil on the authority or recommendation of any one. Would ask Dr. Miner if he had any doubts about the specimens presented being true gall stones?

Dr. Miner: I have doubts about their being true gall stones; they appear to be composed of cholesterine and fecal matter, and were probably formed into this shape in the intestinal canal. Closer investigation should be made before they are pronounced true biliary calculi.

Dr. Miner presented a scirrhus tumor that he had removed from the breast of a woman sixty years of age, and remarked that it looks and feels and has all the characteristics of a cancer. The only remarkable feature about it is, that there is in its centre a distinct bone. This substance has been carefully examined and is undoubtedly true bone. It is remarkable that we should have growth of bone in such locality. The tumor had become so painful and burdensome that removal became necessary. It was external to, and did not involve the mammary gland, but had crowded out the adjacent tissues, and had formed for itself a sort of cyst as is common in such tumors. I believe that it is malignant, and that the bone was developed from the fibrous structure of the tumor.

Dr. Cronyn remarked that these concretions presented by Dr. Gay are not astonishing, and to all appearance are such as are frequently found in the alimentary canal, and probably composed of cholesterine and fecal matter—quite largely of the latter. The tumor presented by Dr. Miner is unusual, perhaps, yet not very strange. Ovarian tumors have been found to contain hairs and other substances that would seem quite as much out of place. This is probably a malignant growth, and the metamorphoses that was the cause of the tumor was sufficient to produce the bone. The subject of tumors is an interesting one, because of their great variety, cause and termination; many that are at first benign become malignant, others presenting in a single tumor three or four distinct characters.

Dr. Miner said that the question of change from benign to malignant disease, was still an open one; pathologists had not yet been able to agree upon this point. It appeared to him as more reasonable to suppose, that the malignant elements of tumors were not discoverable in their early and formative stages, than that they should degenerate into malignant growths after having possessed only the benign elements for a considerable period.

The point of interest and wonder in this case is, that a malignant growth, with tendency to destroy and convert all adjoining tissue into destructive elements like itself, should yet have departed from its usual course, and allowed the formation of any new structure, and especially that of bone.

Miscellaneous business being in order Dr. Miner moved that the Treasurer pay to the Secretary the money necessary to pay the balance on the stove and fixtures. Carried.

Considerable informal discussion was had upon the propriety of appointing a member at each meeting to designate a subject for discussion at the next regular meeting, and prepare himself to lead off in the discussion. All agreed that this course will add much interest to the meetings.

On motion of Dr. Cronyn, Dr. Miner was elected to initiate the course at the next regular meeting.

Adjourned. THOS. M. JOHNSON, Sec'y.

